# A Metabolic Gene Signature to Predict Breast Cancer Prognosis

**DOI:** 10.3389/fmolb.2022.900433

**Published:** 2022-06-29

**Authors:** Jun Lu, Pinbo Liu, Ran Zhang

**Affiliations:** ^1^ Hunan Normal University School of Medicine, Changsha, China; ^2^ Center of Clinical Pharmacology, The Third Xiangya Hospital, Central South University, Changsha, China

**Keywords:** breast cancer, TCGA, metabolism-related gene, survival analysis, prognosis

## Abstract

**Background:** The existing metabolic gene signatures for predicting breast cancer outcomes only focus on gene expression data without considering clinical characteristics. Therefore, this study aimed to establish a predictive risk model combining metabolic enzyme genes and clinicopathological characteristics to predict the overall survival in patients with breast cancer.

**Methods:** Transcriptomics and corresponding clinical data for patients with breast cancer were downloaded from The Cancer Genome Atlas (TCGA) and Gene Expression Omnibus (GEO) databases. Differentially expressed metabolic genes between tumors and normal tissues were identified in the TCGA dataset (training dataset). A prognostic model was then built using univariate and multifactorial Cox proportional hazards regression analyses in the training dataset. The capability of the predictive model was then assessed using the receiver operating characteristic in both datasets. Pathway enrichment analysis and immune cell infiltration were performed using Kyoto Encyclopedia of Genes and Genomes (KEGG)/Gene Ontology (GO) enrichment and CIBERSORT algorithm, respectively.

**Results:** In breast cancer and normal tissues, 212 metabolic enzyme genes were differentially expressed. The predictive model included four factors: age, stage, and expression of SLC35A2 and PLA2G10. Patients with breast cancer were classified into high- and low-risk groups based on the model; the high-risk group had a significantly poorer overall survival rate than the low-risk group. Furthermore, the two risk groups showed different activation of pathways and alterations in the properties of tumor microenvironment-infiltrating immune cells.

**Conclusion:** We developed a powerful model to predict prognosis in patients with breast cancer by combining the gene expression of metabolic enzymes with clinicopathological characteristics.

## Introduction

Breast cancer (BC) has become the most frequent malignancy in women and the primary cause of cancer incidence worldwide (Liu et al., 2021), accounting for 30% of new cancer diagnoses. Although BC mortality has decreased significantly in recent years with advances in cancer treatment, particularly in immunotherapy (Siegel et al., 2018), individual differences in benefits are more pronounced because of tumor heterogeneity and drug resistance (Sharma et al., 2017).

Several cancer hallmarks, such as evasion of growth inhibition and cell death, stimulation of motility and invasion, promotion of angiogenesis, and avoidance of immune destruction, have been linked with metabolism, either directly or indirectly (Hanahan and Weinberg, 2011). Cancer demand causes metabolic alterations in cancer cells relative to non-malignant cells, implying that carcinogenesis and development necessitate the metabolic reprogramming of cancer cells. Metabolic reprogramming is a major hallmark of cancer that promotes cancer cell proliferation, progression, metastasis and resistance to chemotherapeutic agents (Pi et al., 2022). Therefore, focusing on cancer metabolism could substantially advance tumor treatment (Elia et al., 2016). In recent years, with further understanding of the tumor immune microenvironment (TME), increasing studies have focused on the role of metabolism in the TME. Accumulation of abnormal metabolites in the TME has become a hallmark of cancer (Liu et al., 2022). The TME maintains cancer cell proliferation by inducing nutrient removal mechanisms that deplete certain nutrients and force cancer cells to adapt. Previous studies have established that the TME plays a vital role in BC progression. Intermediate metabolism through cell-to-cell interactions in the TME may result in a tumor-suppressive or tumor-promoting phenotype (Dias et al., 2019). A better understanding of the biological pathways of cancer in specific genetic contexts will contribute to the success of targeting cancer metabolism (Vander Heiden, 2011). Therefore, metabolic genes have emerged as promising targets for tumor typing and therapy in many relevant studies. Several metabolic gene signatures have been reported to predict BC prognosis (Gong et al., 2021; Hua et al., 2021; Sun et al., 2021). However, these studies have mainly focused on gene expression data and have not included the influence of clinical factors.

In the present study, we combined gene expression data and clinical information from patients to establish a novel risk prediction model based on the BC (BRCA) data from The Cancer Genome Atlas (TCGA); we then compared the ability to predict the survival of patients with BC compared with that using the conventional tumor, node, metastasis (TNM) staging system.

## Methods

### Data Collection

Transcriptomics and the corresponding clinical data for patients with BC patients were downloaded from TCGA (https://portal.gdc.cancer.gov/) and Gene Expression Omnibus (GEO) (https://www.ncbi.nlm.nih.gov/geo/) databases. In total, 1095 BC cases and 113 adjacent normal cases from TCGA were included in the training set, whereas 327 BC cases from the GSE20685 dataset were included in the test set (patients’ clinical characteristics show in Supplementary Table S1). Clinical data included age, sex, and TNM stage. The Kyoto Encyclopedia of Genes and Genomes (KEGG) PATHWAY database (https://www.genome.jp/kegg/pathway.html) was used to acquire data regarding metabolic enzyme genes (MRG) (Supplementary Table S2). All TCGA and GEO data were obtained from open sources and therefore did not require ethics committee approval. All analyses followed the strict TCGA database access principles and publication guidelines.

### Bioinformatics Analyses

The R packages “limma”, “edgeR”, and “DESeq2” were used, with adjusted thresholds of *p* < 0.05 and |log(fold change) | > 1, to obtain differentially expressed genes from TCGA dataset (Supplementary Table S3). These differentially expressed genes were then intersected with 2891 metabolic enzyme genes obtained based on KEGG PATHWAY. The R packages “survival” and “survminer” were used to run univariate and multifactorial Cox proportional hazards regression analyses, screen and model appropriate biomarkers, and perform test set validation. In the training set, the R package “ClusterProfiler” was used to run KEGG (https://www.kegg.jp/) and Gene Ontology (GO, http://geneontology.org/) enrichment analyses to explore whether there were any differences in biological processes between the high- and low-risk groups. Immune cell infiltration was evaluated using the CIBERSORT algorithm.

### Statistical Analyses

R software (version 4.0.2) was used to perform all the analyses. Before the analyses, all data were log-transformed. Wilcoxon test was used to compare the two groups. Univariate and multivariate Cox regression analyses were used to identify the metabolic enzyme genes linked with overall survival (OS). Kaplan-Meier (KM) survival curve analysis was performed to investigate the differences in survival between the high- and low-risk groups. The sensitivity and specificity of the prognostic model were determined by plotting receiver operating characteristic (ROC) curves.

## Results

### Identification of Differentially Expressed MRGs in BC

**FIGURE 1:**
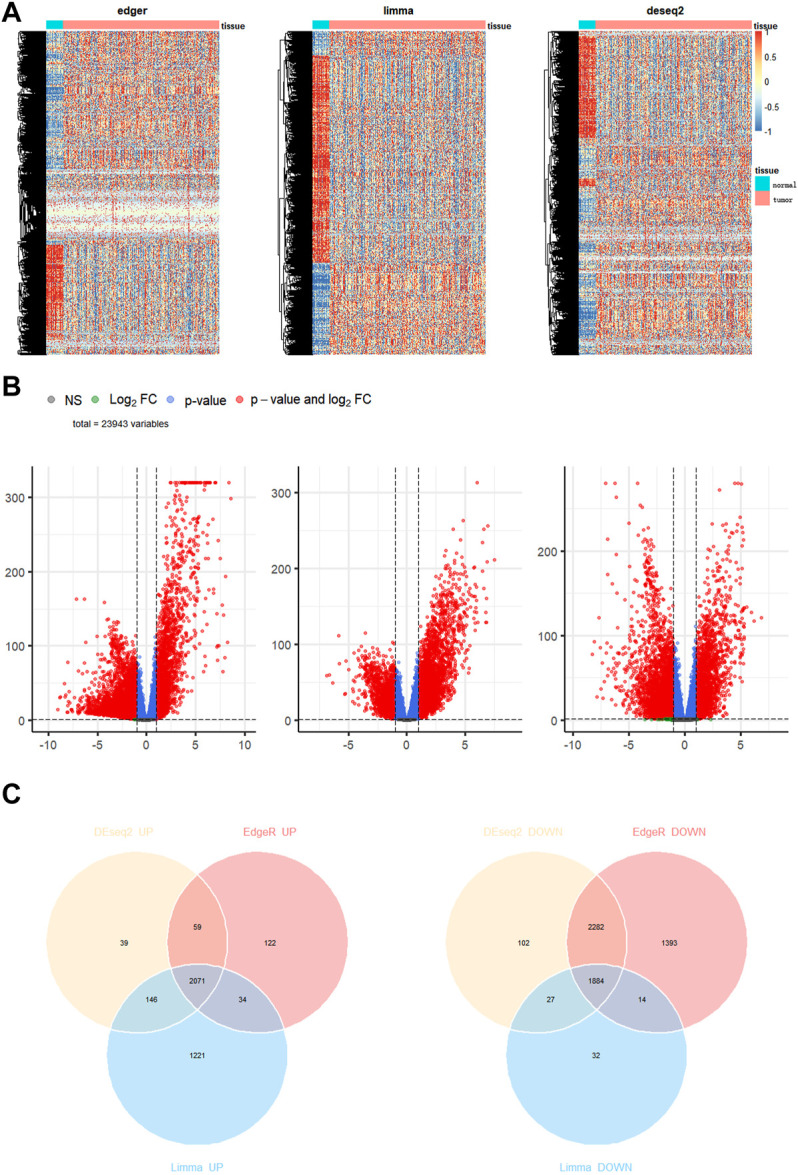
Identification of differentially expressed genes in breast cancer. **(A)** Heatmap **(B)** volcano map **(C)** Venn diagram to compare the results of 3 R packages “DESeq2”, “edgeR”, and “limma”.

We downloaded the gene expression profiles of 113 normal and 1095 BC samples from TCGA. For differential expression analysis in BC and adjacent normal tissues, 2891 MRGs were selected based on the KEGG metabolic pathway-related gene set. Three R packages “DESeq2”, “limma” and “edgeR” were used to screen upregulated differential genes with a threshold *p*-value < 0.05 and |logFC| > 1. After taking intersections with the 2891 MRGs, 212 metabolic genes, which were upregulated in BC, were selected for subsequent analysis. The heatmap, Volcano plot, and Venn diagram of differential genes were drawn using R packages “pheatmap,” “EnhancedVolcano,” and “ggvenn,” respectively (Figures 1A–C).

### Establishment of the Metabolism-Related Gene Prognostic Risk Model in Patients With BC

After identifying differentially expressed genes, we performed modeling. Patients with a follow-up time of 30–3,000 days were included in TCGA data. We initially screened TCGA for 212 differentially expressed MRGs using univariate Cox proportional hazard regression analysis to assess the MRGs related to OS. The results revealed that 29 valid genes were significantly associated with the OS in BC (Supplementary Table S4; *p* < 0.05). We then checked 29 valid genes in the Human Protein Atlas (HPA, https://www.proteinatlas.org/), a database of immunohistochemistry-based expression data for cancer research. Finally, 11 metabolic genes that were significantly overexpressed in BC and associated with survival were identified, of 10 genes (SLC7A5, HPRT1, LPCAT1, SLC12A8, MTHFD2, ALDH18A1, APOO, B4GALT3, ALG3, SLC35A2) that might reduce BC survival were considered risk factors (*p* < 0.05; HR, 1.171–1.681), whereas overexpression of the remaining gene, PLA2G10, may improve survival and was considered a protective factor (*p* < 0.05; HR, 0.8719). The best candidate final regression model for the regression analysis was obtained by screening these 11 genes using the stepwise variable selection procedure in R package, “My stepwise”. Finally, two MRGs, SLC35A2 and PLA2G10, were identified and used to establish a metabolism-related signature. Based on regression coefficients for these two genes in the training set, a model called MGS was built to calculate the risk scores for patients with BC using the following formula: MGS = 0.54527 expression value of SLC35A2 - 0.15133 expression value of PLA2G10.

**FIGURE 2:**
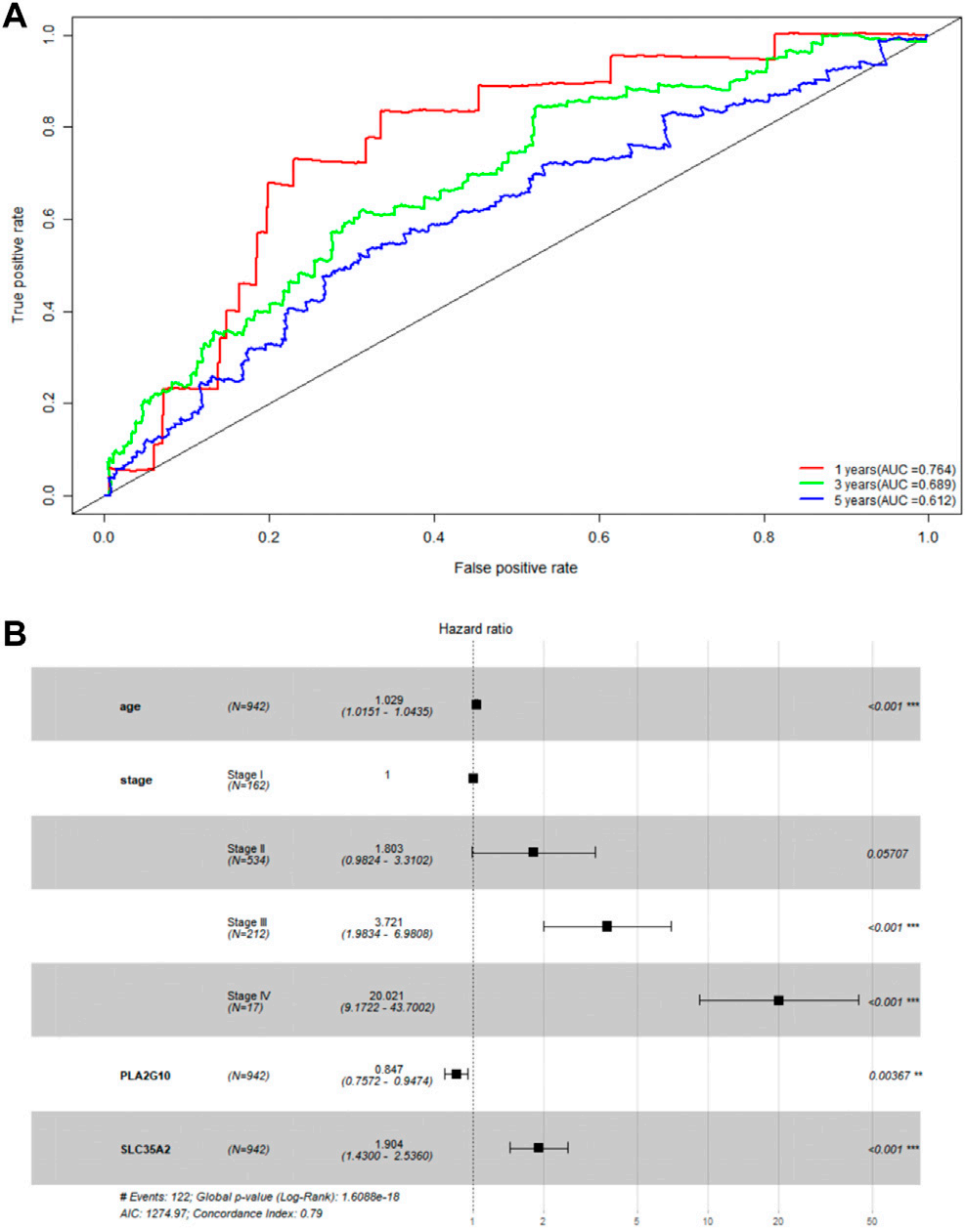
Validation of MGS. **(A)** AUC of time-related ROC curves for MGS at 1, 3 and 5 years in the TCGA cohort. **(B)** Forest map analysis of SLC35A2 and PLA2G10 expression and clinicopathologic characteristics of BC patients by Multivariate Cox Proportional Hazards Regression analysis. *p < 0.05; **p < 0.01; ***p < 0.001.

To initially validate the model, we ran a time-dependent ROC curve analysis on the training set using the R package “survivalROC” and found that the area under the curve was 0.764, 0.689, and 0.612 for 1, 3, and 5 years in the training set, respectively (Figure 2A). We believe that the model has some utility, but the predictions are not as accurate as expected. As covariates including age, sex, and tumor stage can affect the final prediction results as independent variables, we combined age, sex, and TNM staging to build a more accurate model based on the original model. After multivariate Cox proportional hazards regression analysis (Figure 2B), a new predictive model, MAS, was built based on the training set. The TNM staging system was used as a categorical variable, and the formulas used for each stage were as follows: stage I: MAS = 0.02929 × age + 0.64179 × SLC35A2‐0.16310 × PLA2G10; stage II: MAS = 0.02929 × age + 0.64179 × SLC35A2‐0.16310 × PLA2G10 + 0.589644; stage III: MAS = 0.02929 × age + 0.64179 × SLC35A2‐0.16310 × PLA2G10 + 1.32590; stage IV: MAS = 0.02929 × age + 0.64179 × SLC35A2‐0.16310 × PLA2G10 + 3.0152.

### Validation of the Predictive Power of the Prognostic Model

**FIGURE 3:**
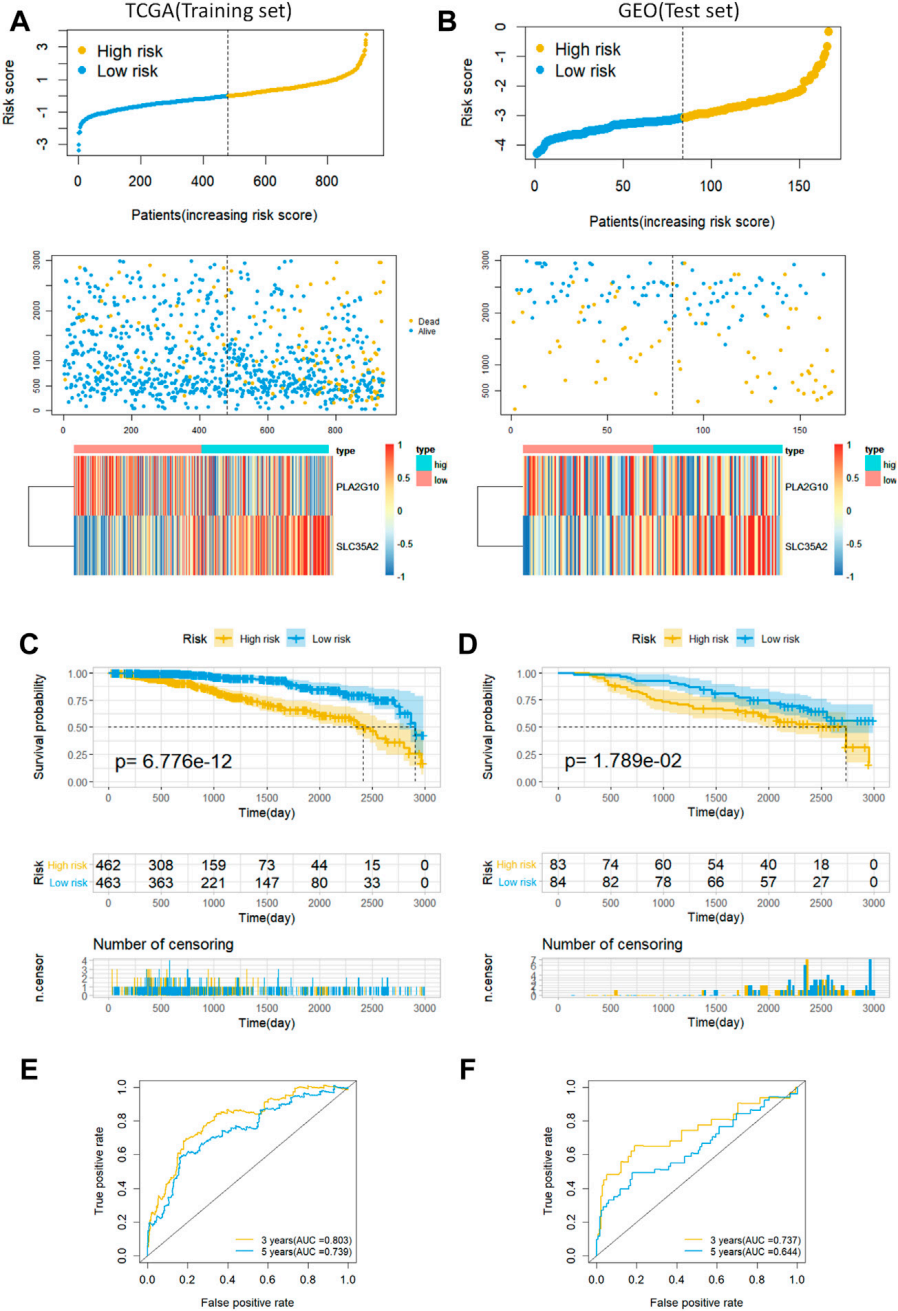
Predictive characteristics of the model MAS in the training and validation sets. **(A,B)** Distribution of risk scores, survival status and heat maps of gene expression for patients in the high- and low-risk groups. **(C,D)** Kaplan-Meier analysis of OS in breast cancer patients based on risk scores. **(E,F)** ROC curve analysis to validate the performance of the MAS in predicting survival at 3 and 5 years for breast cancer.

Next, we validated the prognostic ability of MAS with the training and testing sets. A risk score was first computed for each patient based on the model, and the median risk score was used as the cutoff value to classify patients into low- and high-risk groups. Risk scores, scatter plots, and gene expression heatmaps provided a preliminary picture of the distribution of patients (Figures 3A,B). The Kaplan-Meier log-rank test then revealed that patients in the low-risk group had a much longer OS than those in the high-risk group (Figures 3C,D). Finally, the area under the ROC curve for predicting the 3- and 5-years OS rates was 0.803 and 0.739 in the training set and 0.737 and 0.644 in the validation set, respectively (Figures 3E,F). In addition, we established a nomogram by combining MAS and molecular subtypes of breast cancer (Supplementary Figure S1). These findings indicate that the MAS is a credible model for predicting BC survival.

### GO and KEGG Pathway Enrichment Analyses

**FIGURE 4:**
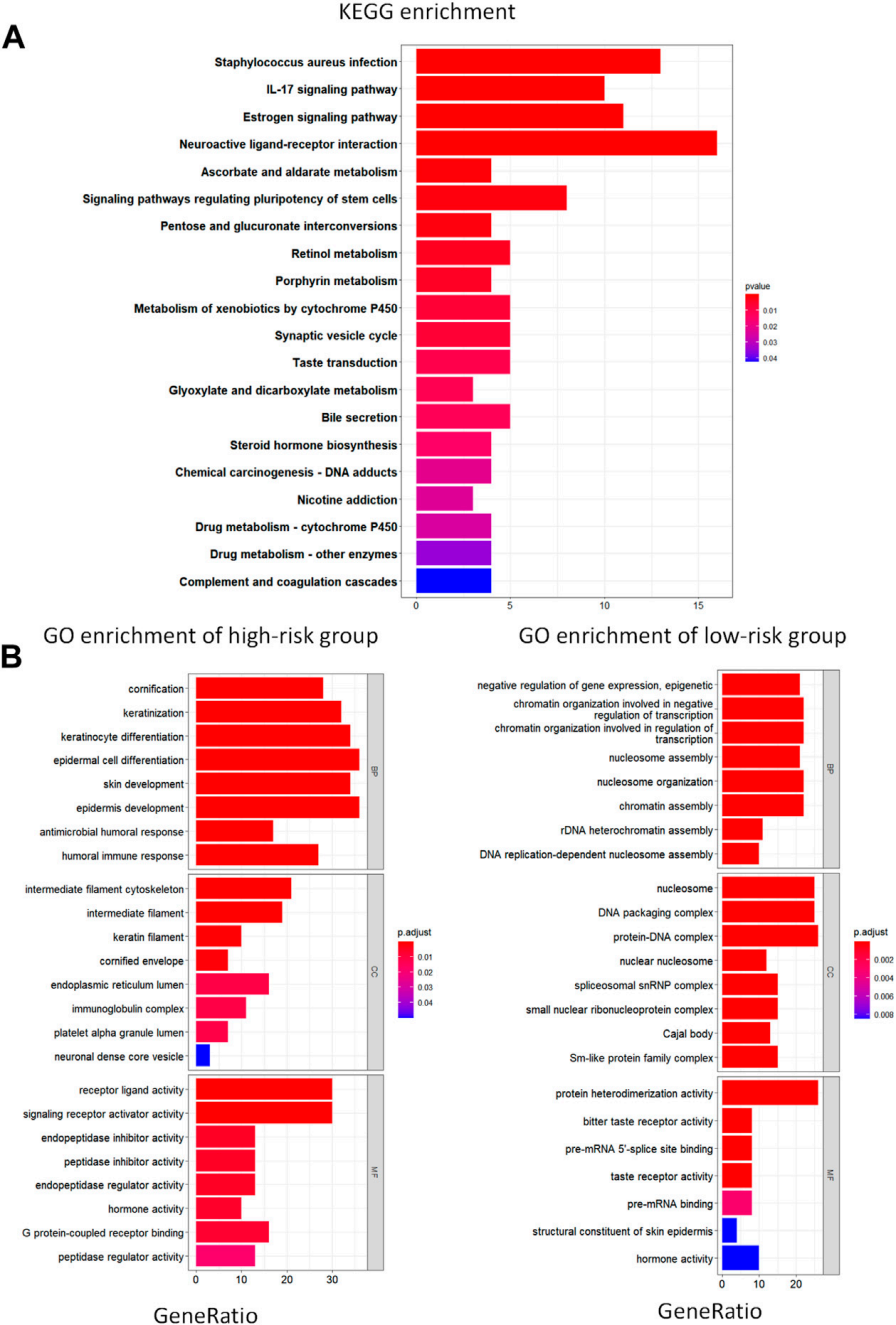
KEGG and GO enrichment analysis. **(A)** KEGG analysis of differentially expressed metabolic genes. **(B)** GO pathway enrichment analysis of differentially expressed metabolic genes for high- and low-risk groups. BP represents biological process, CC represents cellular component and MF represents molecular function.

We used the R package “ClusterProfiler” to perform KEGG and GO enrichment analyses in the training set to investigate whether there were any differences in biological processes between the high- and low-risk groups. With |log2FC| > 1 and FDR 0.05, the R package “EdgeR” was used to identify differentially expressed genes (DEG) in the two groups; we found that the DEG were associated with pathways such as IL-17 signaling pathway, estrogen signaling pathway, and the complement and coagulation cascades (Figure 4A). We demonstrated the expression of these pathways in the high- and low-risk groups based on GO analysis. The epidermal pathway was activated in the high-risk group, whereas pathways related to transcription and the nucleosome were activated in the low-risk group, according to the results (Figure 4B). These discoveries suggest potential mechanisms for different prognoses between high- and low-risk groups.

### Degree of Immune Cell Infiltration in Patients With BC

**FIGURE 5:**
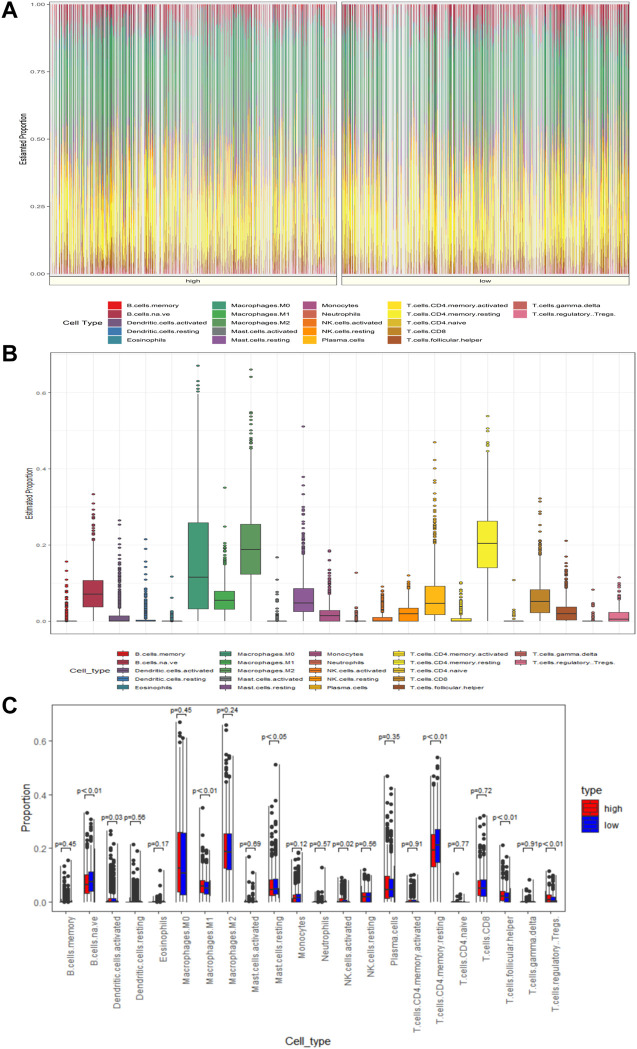
MAS is associated with TME immune cell infiltration. **(A)** Heatmap of the differences in immune cell distribution for each BC patient in low- and high-risk groups. **(B)** Histogram of the distribution of immune cells in all BC patients. **(C)** Differences in the distribution of immune cells in low- and high-risk groups.

The tumor microenvironment plays an important role in tumor development and progression. To assess the relevance of MAS with immune infiltration in the TME, we used the CIBERSOFT algorithm to quantify the comparative composition of multiple immune cell subgroups infiltrating the TME and to compare the abundance of immune cell infiltration in the high- and low-risk groups of BC cases in the training set. Figures 5A,B show the distribution of immune cells in different patient groups. Further the combined difference and correlation analyses revealed that the eight immune cell subgroups exhibited statistically significant differences between the two groups. Among them, M1 macrophages, activated NK cells, follicular helper T cells, and regulatory T cells (Tregs) were positively associated with risk scores. In contrast, naive B cells, activated dendritic cells, resting mast cells, and resting CD4 memory T cells were negatively correlated with the risk scores (Figure 5C). These findings may represent variable levels of immune cell infiltration in the BC TME in high- and low-risk groups.

## Discussion

BC is the most common and fatal cancer in women worldwide and has surpassed lung cancer as the most common cancer according to the global cancer burden data released by the World Health Organization’s International Agency for Research on Cancer (IARC) in 2020. An accurate assessment of the prognosis of patients with BC is essential to ensure appropriate treatment plans. In this study, we combined the clinical information and gene expression data from patients to develop an accurate prognostic prediction model.

In recent years, increasing studies have identified the metabolic activities involved in the progression of cancer and other diseases, which can serve as promising clinical targets for treatment (Pan et al., 2021). Reprogramming energy metabolism is one of the hallmarks of cancer progression (Hanahan and Weinberg, 2011). Metabolic studies on BC tissues and cells have observed changes in metabolic enzymes, fluxes, and mediators, indicating increased glycolysis, TCA cycle activity, glutamine catabolism, and lipid biosynthetic pathways (Dias et al., 2019). The TME plays a determining role in BC progression, whereas intermediate metabolism is actively involved in forming cell-cell interactions in the TME, leading directly to tumor suppressing or promoting phenotypes (Dias et al., 2019). Besides, hypoxia is one of the most typical features of the TME in BC, with HIF-1α activation orchestrating the local disruption of innate and adaptive antitumor immune responses (Malla et al., 2021).

In this study, we established an accurate model for predicting the overall survival of patients with BC using metabolic enzymes and clinical information from TCGA. Using this model, we calculated the risk scores for the patients. Kaplan-Meier analysis revealed that survival in the high-risk groups was lower than that in the low-risk groups. KEGG and GO analyses were used to determine the differences in biological processes. The infiltration of immune cell subpopulations was analyzed by CIBERSORT using the Wilcoxon test to quantify the TME in TCGA BC tissue and to assess the ability of the model for reflecting immune cell infiltration. Our model identified two metabolic enzymes: SLC35A2 and PLA2G10. Mutations in the SLC35A2 gene located on chromosome X induce congenital glycosylation disorders (Hadley et al., 2019). Nucleotide sugars are donors of glycosyltransferases, which are differentially expressed between normal and tumor cells, and their changes can be used to identify relevant BC biomarkers (Scott and Drake, 2019). The role of PLA2G10 in cancer progression has been reported previously. It is upregulated in hepatocellular carcinoma and soft tissue smooth muscle sarcoma (Liu et al., 2007; Tan et al., 2020). The protein encoded by PLA2G10 may contribute to the survival of BC cells via its role in lipid metabolism. However, PLA2G10 was found to have a weak protective effect on the survival of patients with BC in our analysis. Previous findings indicate that patients with BC who are ≤ 40 years old have worse survival and a higher risk of recurrence than that in patients > 40 years old (Cathcart-Rake et al., 2021; Tan et al., 2021). Further, a clinical study showed that the 5-years survival rates of patients with BC in stages I, II, III, IV, and unknown were 100%, 91.9%, 78.8%, 34.2%, and 76.4%, respectively (Mangone et al., 2021). Thus, the prediction model developed using metabolic genes combined with age and the TNM staging system, which are two key clinicopathological characteristics, can provide a more accurate prognostic prediction for patients with BC of different ages and stages.

We performed KEGG enrichment analysis to explore the potential causes of poor prognosis in patients from the high-risk group. The results suggested that the IL-17 signaling pathway was activated in the high-risk BC group, which is consistent with previous reports. IL-17, an important pro-inflammatory cytokine, has been shown to promote proliferation, invasion, and metastasis of BC cells and is significantly associated with poor prognosis in patients with BC (Allaoui et al., 2017). IL-17 promotes the growth of metastatic primary breast tumors by directly promoting tumor cell angiogenesis (Benevides et al., 2015) and indirectly affecting the dependence on neutrophils (Coffelt et al., 2015). In addition, the expression of IL-17 in BC was positively correlated with PD-L1 (Wang et al., 2017)**.** Another pathway that is activated in high-risk patients is the estrogen signaling pathway. Estrogen has been widely reported as a risk factor for many cancer types. In BC cells, estrogen promotes tumor progression by inhibiting apoptosis through upregulated anti-apoptotic Bcl-2 and Bcl-X L (Musgrove and Sutherland, 2009) and activation of MAPK and PI3K/Akt pathways (Gompel et al., 2000). Furthermore, the complement and coagulation cascade pathway, which is upregulated in the high-risk group, has been reported to play an immunosuppressive function in TME, which may be associated with the suppression of anti-tumor CD8^+^ T cell responses (Holers, 2014)**.** The immune infiltration results revealed immunophenotypic differences in the TME between the high- and low-risk groups of patients with BC. Treg infiltration was significantly increased in the high-risk group. Tregs, which are key mediators of immune tolerance, are metabolically reprogrammed in the TME to alter the transcriptional landscape of tumor-infiltrating immune cells, including the transcription factors in tumor-associated macrophages, resulting in the promotion of immunosuppression. Some metabolic proteins have been found to be overexpressed on intratumoral Tregs of patients with BC (Malla et al., 2022)**.** In primary BC, the adaptive immune response of CD4^+^ T cells may often be replaced by immunosuppressive Treg cells, which may induce resistance to checkpoint inhibition (Wesseling-Rozendaal et al., 2022).

Although the prognostic model developed for BC shows strong predictive potential for survival, there are still some limitations to the current study. Our study is limited to a raw analysis using data from publicly available databases, TCGA and GEO. Therefore, additional validation sets are required to verify our conclusions along with experimental validation.

## Conclusion

Overall, we developed and validated a new prognostic model based on metabolism-related genes and clinical data to predict the overall survival in patients with BC. This model can classify patients with BC into two different risk states and reflect the immune status of the TME. Our model may be useful for precise treatment of patients with BC.

## Data Availability

The original contributions presented in the study are included in the article/Supplementary Material, further inquiries can be directed to the corresponding author.

## Abbreviations

BC, Breast cancer; BRCA, breast cancer susceptibility gene; DEG, differential expression gene; GEO, gene expression omnibus; GO, gene ontology; KEGG, kyoto encyclopedia of genes and genomes; MRGs, metabolic enzyme genes; OS, overall survival; ROC, receiver operating characteristic curve; TCGA, the cancer genome atlas; TME, tumor microenvironment; TNM, tumor node metastasis; Tregs, regulatory T cells.
