# Supplementary figures and images for: A Metabolic Gene Signature to Predict Breast Cancer Prognosis
Source: Front Mol Biosci. 2022 Jun 29;9:900433. doi: 10.3389/fmolb.2022.900433 (PMC9277072; doi:10.3389/fmolb.2022.900433)

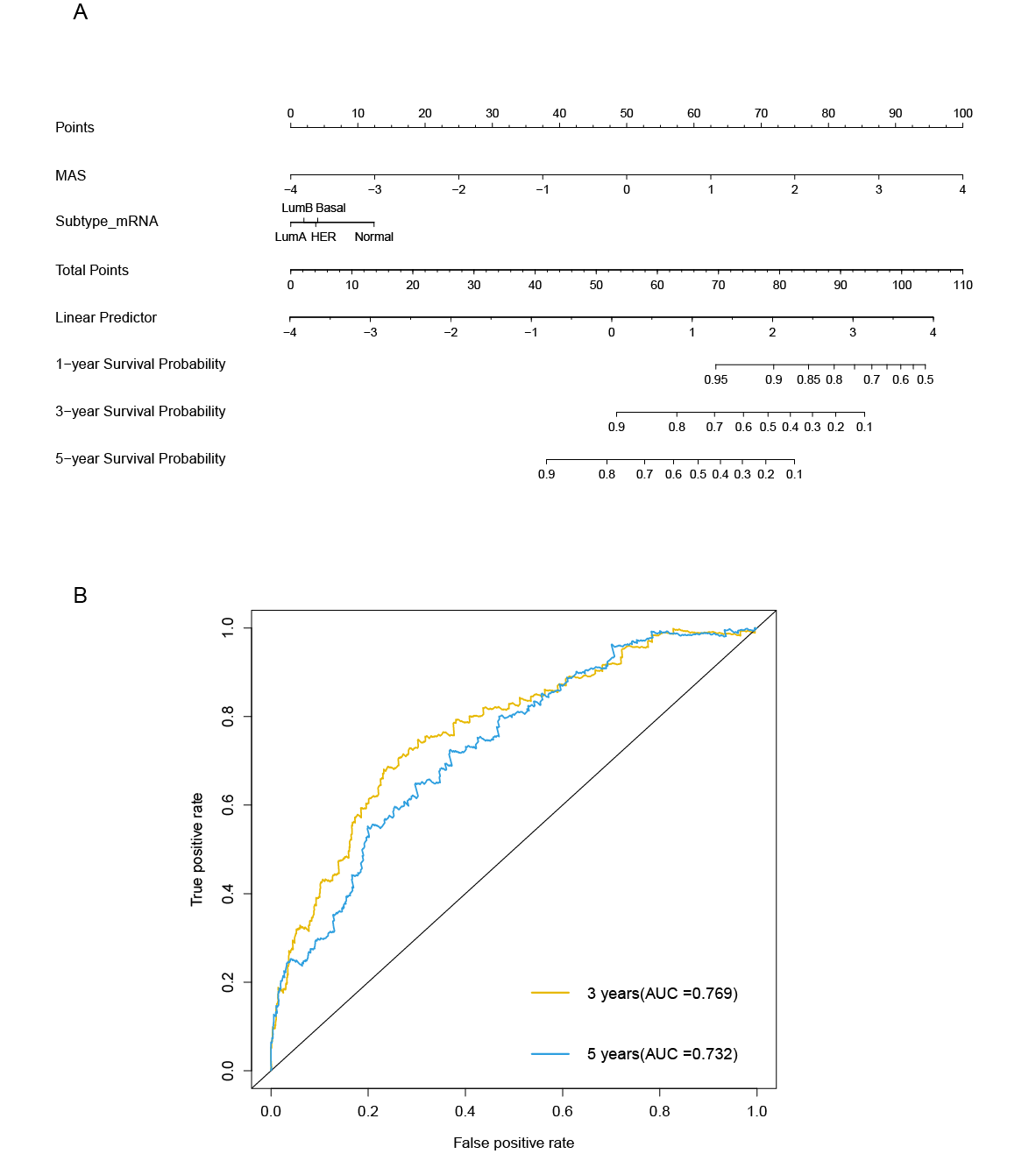

Supplement: Supplementary file 4 [file Image1.tif]
